# Eugenia Jambolana in Diabetes Mellitus

**Published:** 1891-06-06

**Authors:** 


### THERAPEUTICS.

### EUGENIA JAMBOLANA IN DIABETES MELLITUS.

Jambul, an Indian drug derived from the Eugenia Jambolana, Lamark, or Syzigium Jambolanum de Candolle, has been investigated by several observers recently, since the remedy was recommended by Mr. Banatuda in the *Medical Record* in 1883.

In 1887, Scott was struck with the fact that, by the addition of jambul, fermentation could be prevented in a mixture of malt and starch-paste, so that the amount of sugar produced was very much diminished, and his results were confirmed by similar observations made by Balfour and Woodhead. M. Villy has reinvestigated this action, and he found that, as regards this reputed antifermentative action, his results were negative, or, rather, that so far from the saccharine fermentation in mixtures of starch and paste being prevented, they were even accelerated by the presence of jambul.

M. Dujardin-Beaumetz, under whose direction M. Villy's experiments and clinical observations were conducted, has given the powdered seeds in doses varying from three to ten grammes per day, and the results were not altogether uniform. He found that in the severe cases of diabetes, or pancreatic diabetes, the condition of affairs was aggravated rather than relieved. In diabetes of medium intensity, in patients who do not eliminate more than ten grammes of sugar in the twenty four hours, and in conjunction with a scrupulous regulation of diet, jambul appeared to be a favourable adjuvant, contributing to the disappearance of the sugar. But if the patient began to take starchy food again, or relaxed his dietetic *régime*, the condition of the patient became as bad as ever. He asserts that jambul can be of service if administered with the proper precautions as to diet. M. Duboisquet-Laborderie, at the meeting of the Société de

Thérapeutique, February 11th, 1891, stated that he could not confirm the opinions of M. Dujardin-Beaumetz and M. Villy. He had made trials of jambul since the month of June, 1890, and had prescribed it in five cases, with the results, briefly indicated, as follows: (1) The patient was a vigorous man of 65, of the arthritic temperament. Five years previously he had examined his urine during an attack of pneumonia, and found it contained 150 grammes of sugar in two litres of urine in the twenty-four hours. He was convinced that this patient had been suffering from diabetes for a long time without knowing it. With strict dieting and antipyrin (prescribed by M. Dujardin-Beaumetz) for three years, the amount of sugar did not exceed eight or ten grammes in twenty-four hours, and during this period he was sometimes free from sugar for five or six days at a time. Last July he gave this patient jambul in two-gramme doses. During the first day or two the sugar was increased to about thirteen grammes, but it disappeared altogether about the fifth day. For more than twenty days his urine was free from sugar, and then he left off the jambul, and he returned to his ordinary condition. His patient, whether he owed his remarkable amelioration to jambul, or whether he had taken a favourable turn, passed no more sugar for eight or ten days, although he had stopped all medication, and with it the dietetic and hygienic measures recommended to diabetics. (2) An arthritic female patient, at the commencement of her illness, three years before, the amount of sugar was 50 grammes in 3 litres of urine in twenty-four hours. In spite of a rigid adherence to restricted diet, the sugar never entirely disappeared, and even under these conditions, and with the use of antipyrine averaged 6 or 7 grammes daily. Jambul caused the sugar to disappear altogether in six or seven days, and at the same time the amount of urine passed fell to 1,500 to 1,800 grammes instead of two to two and a-half litres. (3) A man of 65 years came under his notice in a very advanced cachectic condition—since dead from a very rapid form of tuberculosis—passed four litres of urine daily, with 75 grammes of sugar on the average. The results of administering jambul were *nil*. (4) A strong man of 50 years, a wine merchant, with a history of alcoholism, in whom after some weeks of rigid dieting, jambul produced a fall in sugar from a daily average of 35 grammes to three or four grammes. He continued to take jambul, but he has gone back to his old habits, and abandoned his exclusive diet, and has returned to his former condition. (5) In a very bad case (100 grammes of sugar daily), in spite of all dietetic and hygienic precautions, jambul gave no results. Contrary to the opinions of foreign practitioners, who have stated that jambul causes sugar to disappear without any special dieting, in his three cases in which jambul had given good results, he had found that the slightest departure from restricted diet caused the sugar to reappear as before, or even to be increased. Under the influence of jambul, together with a rigid dieting, which condition was a *sine quâ non*, sugar disappeared from the third to the fifth day in mild diabetic cases, or those of medium severity, but he did not seem to get any result in bad cases. The sugar reappeared on the cessation of the drug, to which the patient became accustomed very soon. With regard to the generally-received opinion that the seeds alone gave the best effect, M. Laborderie has only utilised the powdered bark, and the results seemed identical with the observations of MM. Dujardin-Beaumetz and Villy. He administered it in doses of two to ten grammes, and never saw any bad results, with the exception of his first patient, who had diarrhœa, with a tendency to vomiting. But this patient had previously had two attacks of severe gastro-enteritis, and his stomach and intestines were therefore unusually susceptible. The powdered jambul in combination with opium was then always well supported. He has also found a tendency to constipation in the patients, due to the astringent properties of the drug. This fact, which has been observed by others, led him to administer it in cases of diarrhœa.

M. Villy, to whose investigations we have already made allusion, has devoted his inaugural thesis in Paris this year to a study of the therapeutic value of jambul in diabetes. His results may be summarised as follows: (1) Jambul should not be used in diabetes if the ordinary diet is continued; under such conditions, both glycosuria and the formation of urea increase instead of diminishing. (2) In slight cases, with or without increased nitrogenous excretion, jambul in moderate doses may be employed with benefit, always, however, insisting on a regulated diet. This latter point is absolutely necessary, and under these conditions the sugar will soon, in many cases, disappear, to return, however, on discontinuing the drug. It is to be feared, too, that tolerance will soon be set up, when the effect will, of course, be lost. (3) In severe cases the effect is entirely negative. As soon as a regulated diet appears powerless, all adjuvants—jambul among the number—are unavailing. These results apply, of course, only to the effects of the dried and powdered seeds; whether the fresh plant is more efficacious experiments in India will alone show.

Further, we must refer to the reports of Professor Lewaschew, of Kasan, who has used the drug during the last two years, and has had very favourable results. He has used it in eight cases, and the one given may be taken as an example of the rest. This patient, a man aged 62, had suffered from diabetes for five years. When admitted to the Kasan University Clinic, he was much emaciated and very weak, and suffered greatly from thirst. The quantity of urine passed in the twenty-four hours was 8,380 cubic centimetres, its specific gravity was 1028, and it contained sugar to the amount of 688 grammes (8·3 per cent.). The body weight was 112 lbs. Jambul was given in the form of powder, the daily dose being four grammes; the seeds used for this purpose were old and mouldy. No other treatment was employed, and the patient was allowed to take his usual diet, which included abundance of sugar with bread and other amylaceous substances. The dose was gradually increased to 30 grammes a-day, and after a fortnight the medicament was given in the form of a fluid extract from the same seeds. As no effect whatever was produced the treatment was discontinued. Some time afterwards a supply of fresh seeds was obtained, and powder made from them was exhibited in daily doses of 20 grammes. This time "an immediate very marked effect" was observed. Four days before the commencement of the treatment with the fresh jambul was begun, the daily excretion of urine was 11 litres, the amount of sugar being 777 grammes; on the day following the administration, both urine and the sugar were less, and in three days the diminution was very considerable. The body weight rose to 116½ lbs., and the man gained strength. For twelve days he took 20 grammes of the powder, then for three days 25, and finally 30 grammes for eight days. At this time the amount of urine had fallen to 3·800 grammes; the weight was 118 lbs. The treatment was then discontinued for a time, when at once the disease began to make headway again, the urine increasing to 10½ litres and the sugar to 840 grammes, while the weight fell to 115½ lbs. A further course of the powder (25 grammes a day for eight days) speedily enabled the patient to recover the ground he had lost. When discharged the amount of urine was 3,100 grammes, containing 211 grammes of sugar. When seen again a month later, during which time the treatment had been discontinued, the amount of urine was found to vary from 6,080 to 7,050, and that of the sugar from 455 to 547·5 grammes a day. In all other cases similar effects were noticed, so that Lewaschew feels himself justified in concluding that when jambul is used in sufficient doses (from 20 to

40 grammes a day) in diabetes mellitus, diminution in the amount of urine and sugar, and marked improvement of the symptoms, take place. The amelioration lasts for a longer or shorter period of time after the discontinuance of the treatment. He has not yet seen complete disappearance of the sugar in any case. He never observed any unfavourable secondary effects from the administration of jambul.

Dr. Wilde in the *Medical Annual* for 1888 warns us not to confuse jambul seeds with "jumble beads" or jequirity seeds. He further refers to Dr. Kingsbury's favourable reports of a case of diabetes in which jambul was very useful. The patient was suffering from great thirst and ravenous appetite; there was also great restlessness. The urine had a sp. gr. of 1,040 to 1,042, and from 7 to 7½ quarts were passed daily. Five grains of jambul were given six times in the twenty-four hours for a fortnight. At the end of that period the patient was able to get up and walk out for an hour at a time, was neither thirsty nor abnormally hungry, and was passing four to five quarts of urine of a specific gravity of 1,020; he could sleep well, and felt strong. During the time he was taking the jambul his diet was not restricted in any way. Mr. E. Hurry Fenwick has reported some very successful cases treated with this remedy.* We have used jambul in cases of diabetes during the last two years with favourable results, and we have referred at some length to the reports of several observers of this remedy, because the drug does not seem to have met with the reception it deserves. The references to cases will serve to indicate the class of case in which jambul may be expected to render service and the restrictions to be observed in administering it.

